# Evaluation of salivary pH and buffering capacity in gastroesophageal reflux among patients with dental erosion

**DOI:** 10.6026/973206300214842

**Published:** 2025-12-15

**Authors:** Shweta V Sagare, Jasmine Marwaha, Vidya Nagabhushan, Deepankar Dass, Mohd Ahmed Ali khan, Roger Biswal

**Affiliations:** 1 Department of Conservative Dentistry and Endodontics, Bharati Vidyapeeth (Deemed To Be University) Dental College and Hospital Sangli, Maharashtra, India; 2 Department of Conservative dentistry and Endodontics, National Dental College and Hospital, Derabassi,140507, India; 3 Oral Pathologist, Pandits dental care, LIC colony srirampura 2nd Stage, Mysore-570023, Karnataka, India; 4 Department of Conservative Dentistry & Endodontics, Maharshi Markendshwar College of Dental Sciences & Research, Mullana, Haryana, India; 5 Department of Conservative dentistry and endodontics, Rungta college of dental science and research, Chattisgarh; 6 Department of Oral Medicine and Radiology, Kalinga Institute of Dental Sciences, KIIT Deemed To Be University, Patia, Bhubaneswar, Odisha, India

**Keywords:** Gastroesophageal reflux disease, salivary pH, buffering capacity, dental erosion, tooth wear, saliva, BEWE index

## Abstract

Gastroesophageal reflux disease (GERD) places the mouth tissues under the acidic effects of the gastrointestinal secretions, which may affect the protective effects of salivation and increase the susceptibility of the dentures. Therefore, it is of interest to compare salivary pH, buffering capacity and dental erosion among 45 GERD patients and 45 controls. The patients with GERD demonstrated a large reduction in salivary PH and buffering capacity, and dental erosion was much more prevalent. The salivary parameters were found to be negatively correlated with the erosion severity. The impact of GERD-related changes in saliva is a significant factor in the case of dental erosion, which justifies the significance of interdisciplinary prevention and management approaches.

## Background:

Gastroesophageal reflux disease (GERD), is an extremely widespread chronic illness which is associated with retrograde movement of gastric content into the esophagus and it is said to have afflicted about 13-20% of the world population [1]. In addition to the usual esophageal complications such as heartburn and regurgitation, GERD is also being known to have extra-esophagic complications, especially oral ones, as well [2]. Explicit oral tissues to acidic gastric material with pH levels down to 1.0-2.0 represent major difficulties to the hard tissues of the denture [3]. Dental erosion, which is the permanent loss of dental hard tissue by non-bacterial chemicals, is one of the significant oral health issues in GERD patients [4]. The dental erosion has been shown to be prevalent in GERD populations between 24% and 95% which is significantly more than that of the general population [5]. The acid demineralization of enamel and dentin is the main erosive agent that may trigger the progressive erosion of the tooth, which may undermine the functioning, aesthetics, and quality of life [6]. Saliva is a vital protective agent against dental erosion by several mechanisms such as dilution and clearance of acids, neutralization of acids by buffering systems and supplying calcium and phosphate ion to remineralize teeth [7]. Saliva pH is a critical factor influencing saliva protective capacity with the lower pH values being linked to a decrease in neutralizing capacity and an increase in demineralizing capacity [8]. Regular resting salivary pH is between 6.5 and 7.5 and the enamel dissolution critical pH is about 5.5 [9]. The capacity of saliva to oppose pH changes when in the presence of acids is called salivary buffering capacity and is mainly due to the bicarbonate, phosphate, and protein buffer systems [10]. Sufficient buffering capacity will be necessary to ensure oral pH homeostasis and prevent dental tissues against the erosive challenges [11]. It has been recommended that such people with impaired buffering ability might be predisposed to dental erosion despite the same acid exposure [12]. Past studies have shown salivary changes of composition and flow rate among GERD patients [13, 14]. Nevertheless, the nature of the relationship between the salivary buffering capacity and the degree of dental erosion of GERD patients is not fully described. Moreover, although some studies have conducted researches on individual salivary parameters of GERD patients, there are very few research works, which evaluate both pH and buffering capacity with a systematic evaluation of dental erosion [15]. Clinical significance of the study on the interaction between GERD-induced salivary pH changes and dental erosion is the ability to develop prevention strategies and recognize high-risk patients who could be assisted by more effective measures on dental monitoring and protection [16]. Also, salivary parameters can be applied as effective biomarkers of oral complications in GERD and direct specific interventions [17]. Although the awareness of the oral manifestation of GERD is increasing, there are still knowledge gaps in the impact of chronic exposure to acid on salivary protective processes, and the correlation of such changes with clinical patterns of erosion. Majority of the available literature has been done on the Western population with little information on the various demographic groups. In addition, salivary parameters could serve as potentially valuable predictor variables of erosion risk in GERD patients, and thus require further research [18]. Therefore, it is of interest to assess and compare the salivary pH and buffering capacity of gastroesophageal reflux disease patients and healthy controls and evaluate the relationship between these salivary parameters and the incidence and severity of dental erosion.

## Materials and Methods:

Sample size was calculated using G*Power software (version 3.1.9.7) based on anticipated mean difference in salivary pH of 0.45 units with a pooled standard deviation of 0.60, α = 0.05, and power of 0.85. This yielded a minimum requirement of 38 participants per group. To account for potential dropouts and incomplete data, 45 participants were recruited for each group, totaling 90 participants.

## Participant selection:

## Inclusion criteria for GERD group:

[1] Age 25-60 years

[2] Confirmed GERD diagnosis by gastroenterologist based on clinical symptoms and esophagogastroduodenoscopy (EGD) findings

[3] Minimum disease duration of 6 months

[4] Active symptoms (at least 2 episodes per week)

[5] No change in GERD medication in the previous 3 months

## Inclusion criteria for control group:

[1] Age 25-60 years

[2] No history of GERD or chronic acid reflux symptoms

[3] No current use of proton pump inhibitors or H2 receptor antagonists

[4] Matched to cases by age (±3 years) and gender

## Exclusion criteria for both groups:

[1] Current smokers or tobacco users

[2] Pregnancy or lactation

[3] Systemic diseases affecting salivary gland function (Sjögren's syndrome, diabetes mellitus, rheumatoid arthritis)

[4] Head and neck radiation therapy history

[5] Medications known to affect salivary flow (anticholinergics, antidepressants, antihistamines)

[6] Current orthodontic treatment

[7] Recent (within 3 months) professional fluoride application

[8] Eating disorders (bulimia nervosa, anorexia nervosa)

[9] Professional exposure to acids (swimmers, industrial workers)

[10] Xerostomia or salivary gland disorders

## Clinical Examination and GERD Assessment:

All participants underwent comprehensive medical and dental history recording. GERD patients were classified by disease severity using the Los Angeles Classification based on EGD findings. GERD duration was documented from the time of initial diagnosis. Current medications, including proton pump inhibitors (PPIs) and H2 receptor antagonists, were recorded.

## Dental erosion assessment:

Dental erosion was evaluated by a single calibrated examiner (intra-examiner reliability: kappa = 0.88) using the Basic Erosive Wear Examination (BEWE) index. The oral cavity was divided into sextants, and each tooth was examined under standardized dental unit lighting after professional cleaning and air-drying.

The BEWE scoring system was applied as follows:

[1] Score 0: No erosive tooth wear

[2] Score 1: Initial loss of surface texture

[3] Score 2: Distinct defect, hard tissue loss <50% of surface area

[4] Score 3: Hard tissue loss ≥50% of surface area

The highest BEWE score in each sextant was recorded, and the cumulative score (sum of all sextants, range 0-18) was calculated for overall erosion severity classification.

## Saliva collection:

Saliva samples had been collected at 9:00-11:00 AM to reduce circadian effects. The participants were asked to avoid eating, drinking or chewing gum, as well as undergoing any kind of oral hygiene just 2 hours before collection. Before collecting them, they rinsed their mouths with water 10 minutes beforehand.

## Unstimulated whole saliva:

The participants were sitting upright with their head tilted towards the front. They were instructed to sacrifice 60 seconds of not swallowing the saliva in the mouth, and to then make expectoration in a graduated sterile test tube. This procedure was done 5 minutes and total volume was measured.

## Stimulated whole saliva:

The participants were chewing a standardized piece of paraffin wax in a rate of around 70 chews per minute. The same expectoration method was used to collect saliva during 5 minutes. All the samples were put on ice and subjected to processing less than 30 minutes after collection.

## Salivary pH Measurement:

The calibrated digital pH meter (Mettler Toledo SevenCompact S220, Switzerland) with the resolution of 0.01 pH units was used to measure the salivary pH. The measurement session was preceded by the pH meter calibration using the standard buffer solutions (pH 4.0, 7.0 and 9.0). They were measured at room temperature (25 C) and the electrode was completely immersed in 2mL of saliva. It was noted at the time of stabilization of the value of the reading (±0.02 pH units in 30 seconds). Measurement of each sample was done twice and the average taken and analyzed.

## Buffering capacity assessment:

Salivary buffering capacity was assessed on the basis of the modified Ericsson. In short, 1 mL of stimulated saliva was placed in a test tube of 3 mL of 0.005 N hydrochloric acid (HCl). The mixture was stirred with the help of a mechanical shaker within 20 minutes and the final pH was recorded with the help of the calibrated pH meter.

## Buffering capacity was rated as:

[1] High: Final pH ≥ 6.0

[2] Medium: Final pH 4.5-5.9

[3] Low: Final pH < 4.5

## Statistical analysis:

The SPSS Statistics 27.0 (IBM, Armonk, NY, USA) was used to analyse the data. Shapiro-Wilk tests and Q-Q plots were used to test normality. Continuous variables were normally distributed and were indicated as mean and standard deviation and their comparison was done using independent t-tests. Measures of categorical variables were in terms of frequencies and percentages, and compared by chi-square or by Fisher exact test depending on the situation. To determine the correlation between continuous variables, Pearson correlation coefficients were used. The independent predictors of the degree of dental erosion were analyzed using multiple linear regression analysis with BEWE cumulative score serving as a dependent variable. The level of statistical significance was defined to be p<0.05.

## Results:

A total of 90 participants were enrolled and completed the study: 45 GERD patients (24 males, 21 females; mean age 42.8 ± 9.6 years) and 45 healthy controls (24 males, 21 females; mean age 41.6 ± 9.2 years). There were no significant differences in age (p=0.538) or gender distribution (p=1.000) between groups. Among GERD patients, the mean disease duration was 4.7 ± 2.8 years (range 0.5-12 years). According to Los Angeles Classification, 15 patients (33.3%) had Grade A, 18 (40.0%) had Grade B, 9 (20.0%) had Grade C, and 3 (6.7%) had Grade D disease. Forty patients (88.9%) were using proton pump inhibitors, and 5 (11.1%) were using H2 receptor antagonists. Table 1 (see PDF) presents the comparison of salivary parameters between GERD patients and controls. GERD patients demonstrated significantly lower unstimulated salivary pH (6.32 ± 0.48) compared to controls (6.85 ± 0.38; mean difference -0.53, 95% CI: -0.71 to -0.35, p<0.001). Similarly, stimulated salivary pH was significantly reduced in GERD patients (7.12 ± 0.52) versus controls (7.58 ± 0.41; mean difference -0.46, 95% CI: -0.65 to -0.27, p<0.001). Regarding buffering capacity distribution (Table 2 - see PDF), the GERD group showed a significantly different pattern compared to controls (p<0.001). In the GERD group, 28 patients (62.2%) had low buffering capacity, 13 (28.9%) had medium buffering capacity, and only 4 (8.9%) had high buffering capacity. In contrast, the control group had 10 participants (22.2%) with low buffering capacity, 17 (37.8%) with medium buffering capacity, and 18 (40.0%) with high buffering capacity. Dental erosion was significantly more prevalent in GERD patients (82.2%, 37/45) compared to controls (28.9%, 13/45; p<0.001). The mean cumulative BEWE score was markedly higher in GERD patients (6.4 ± 4.2) compared to controls (1.2 ± 1.8; p<0.001). According to BEWE risk classification, 10 GERD patients (22.2%) had high-risk scores (9-18), 18 (40.0%) had medium-risk scores (3-8), 9 (20.0%) had low-risk scores (1-2), and 8 (17.8%) had no erosion. In the control group, no participants had high-risk scores, 3 (6.7%) had medium-risk scores, 10 (22.2%) had low-risk scores, and 32 (71.1%) had no erosion. Among GERD patients with erosion, the most commonly affected sites were palatal surfaces of maxillary anterior teeth (78.4% of patients with erosion) and occlusal surfaces of mandibular molars (67.6%). The distribution of BEWE scores by tooth surfaces showed that 43.2% of all erosive lesions were scored as grade 2 (distinct defect), 38.9% as grade 1 (initial surface loss), and 17.9% as grade 3 (severe loss). Table 3 (see PDF) presents the correlation analysis between salivary parameters, GERD characteristics, and dental erosion severity. Cumulative BEWE score showed significant negative correlations with unstimulated salivary pH (r=-0.614, p<0.001), stimulated salivary pH (r=-0.582, p<0.001), and final pH after HCl challenge (r=-0.558, p<0.001). GERD duration showed a significant positive correlation with BEWE score (r=0.521, p<0.001). Unstimulated and stimulated flow rates showed moderate negative correlations with erosion severity (r=-0.387, p=0.001 and r=-0.412, p<0.001, respectively).

The results were analysed using multiple linear regression, and cumulative BEWE score was the dependent variable. The variables included in the model were GERD status, unstimulated salivary pH, buffering capacity (end pH after HCl), GERD duration, age, and gender. This model was important (F=42.6, p<0.001) and it explained 78.4% of the severity in erosion (adjusted R 2=0.784). The independent predictors were: GERD presence (=3.12, = -2.24, 95% CI: -3.18 to -1.30, =0.001), unstimulated salivary pH ( = -2.24, = -1.86, 95% CI: -2.64 to -1.08, =0.002), buffering capacity ( = -1.86, = -2.24, 95% CI: -2. Patients of the GERD group with low buffering capacity scores (8.2 +/-3.6) tended to have mean BEWE scores that were significantly higher than those of the medium (4.8 +/- 2.9; p=0.009) and high-buffering capacity groups (2.5 +/- 1.3; p=0.003). In the same way, the patients of GERD whose unstimulated salivary pH was below 6.0 reported better BEWE scores (9.1 ± 3.8) than those whose salivary pH was above 6.0 (4.6 ± 3.2; p<0.001).

## Discussion:

This case-control study established that patients with GERD have highly impaired salivary pH and buffering power as compared to healthy controls, and these changes have a very close relationship with prevalence and severity of dental erosion. Our hypothesis is proved and quantitatively presents the role of salivary dysfunction in oral complications of GERD. The high discrepancy in the difference between unstimulated and stimulated salivary pH in the GERD patients is consistent with the past studies. The pathways of lowered salivary pH in GERD are complex. The sustained intake of regurgitated gastric acid directly decreases the oral pH and even the short-term exposure to gastric content with a pH of 1.0-2.0 may overwhelm the salivary buffering mechanisms [19, 20]. Moreover, GERD can also regulate the functioning of salivary glands due to vaginal or inflammatory mechanisms, which can influence the secretion of bicarbonate, which is the main buffer in saliva [21]. The fact that 62.2 percent of GERD patients exhibited low buffering capacity as compared to the 22.2 percent of controls is a crucial observation with far reaching clinical implications. Salivary buffering capacity is necessary in neutralizing acids and pH homeostasis [22]. Buffering disability in GERD patients poses a situation of a double jeopardy: the acid is more exposed, and the neutralizing ability is less than usual [23]. The large rate of dental erosion observed in GERD patients (82.2% vs. 28.9% in controls) supports the findings of systematic reviews which indicate prevalence of dental erosion of between 24 to 95 percent in GERD populations [24]. The significant difference in the mean BEWE scores (6.4 vs. 1.2) implies that in patients with GERD, there is an increased prevalence and severity of erosive lesions. The tendency of palatal surfaces of maxillary anterior teeth to be preferred during regurgitation corresponds to the common sequence of erosion during regurgitation, in which gastric material initially touches the palatal surfaces [25]. The quantitative support of the protective effect of saliva is represented by the high negative Coefficients of salivary pH, buffering capacity and the severity of dental erosions (r=-0.614 and r=-0.558, respectively). These correlations are higher as compared to some of our past research which may be because of our standardized measurement protocol and controlled participant selection [26]. The multiple regression analysis also showed that salivary parameters are independent predictors of the extent of erosion, although the presence and duration of GERD were controlled. This implies that the differences in salivary activity are a strong individual contributor of the erosion risk in GERD patients. The fact that the duration of GERD was positively correlated with the severity of erosion (r=0.521) is an indication of a cumulative effect of chronic acid exposure. In a similar manner, the correlation with the severity of GERD (Los Angeles grade) implies that patients with a milder esophageal inflammation have more oral complications, which is probably because of the higher number of reflux events and higher volume of the same [27]. These results highlight the value of proper GERD treatment not only to relieve the symptoms of the gastrointestinal tract but also avoid the development of dental defects. Remarkably, even in the group of GERD patients, the ones whose salivary function was not completely destroyed (greater pH and buffering capacity) exhibited much less erosion. This observation implies that the salivary protective measures should be used to reduce the risk of erosion among GERD populations. Probably, some approaches to pursue are saliva stimulation, buffering agent utilization, and optimization of GERD drug regimens [28]. Further, the statistically significant low salivary flow rates in the GERD patients were not as low as the difference in pH and buffering capacity. This indicates that qualitative changes in salivary might be significant than quantitative changes in erosion related to GERD. Nevertheless, both flow rates and severity of erosion are significantly correlated with one another, which imply that the two factors are the contributors of overall risk [29]. Clinically, the findings affirm the inclusion of dental assessment into the GERD management protocols. Gastroenterologists must be informed about the risks of oral complications and the need to refer the patient to the dentist, especially when the disease is chronic or severe [30]. On the other hand, the dentists who detect the presence of unexplained erosive tooth wear should screen patients with GERD symptoms and consider medical referral. Salivary tests, especially pH and buffering capacity, could serve to detect the patients at high risk, and subject them to intensive preventive treatment [31]. The preventive measures to be provided to the GERD patients include dietary counseling to reduce the further exposure of the acid to erosive food and beverages, fluoride products to increase the process of remineralization, buffering or neutralizing rinse to be used after the incidences of reflux, and optimization of oral hygiene timetable to reduce brushing right after exposure of acid [32]. Furthermore, GERD could be effectively managed through medical intervention by use of proton pump inhibitor or surgical procedures to lower the risk of oral acid exposure, although our data revealed that, most of the patients under proton pump inhibitor (PPI) medical management still developed erosion, indicating that the present trend in managing GERD may not offer complete protection against oral complications [33]. Some of the strengths of this study are that the participants have been carefully selected with clear inclusion/exclusion criteria, standard protocols have been used to collect and measure saliva, valid history of assessing erosion using BEWE and a thorough statistical analysis including multivariate modeling. There should, however, be some limitations. The cross-sectional design does not allow causality and evaluation of time relations. The dietary and extrinsic acid exposures were not evaluated quantitatively, but patients with eating disorders and professional exposure to acid were excluded. The research was performed in one centre, which might be a limitation to generalizability. Also, other salivary elements like the calcium, phosphate, and protein concentration were not measured; these also play a protective role [34]. The longitudinal designs in the future research should be utilized to monitor salivary changes and erosion development in the GERD patients over the course of time, interventions to improve salivary protective capacity, the genetic factors that affect the individual susceptibility, the complete salivary composition including minerals and proteins, the effects of the various GERD treatment modalities on the outcome of the oral cavity [35].

## Conclusion:

The salivary pH and buffering capacity is much lower in GERD patients and thus increases the prevalence and severity of dental erosion. Salivary parameters are important predisposing factors of erosion that underscores the essential protective nature of saliva against tooth erosion caused by acid. There should be integrated medical and dental care to prevent and check oral problems in GERD patients.
